# Draft genome sequence of *Kosakonia oralis* 282A-S69, a novel member of the genus *Kosakonia* isolated from the human oral cavity

**DOI:** 10.1128/mra.00075-26

**Published:** 2026-04-20

**Authors:** Reyaz Ahmad Khan, Madhusmita Bhagawati, Robinson C. Jose, Rajeev Sarmah

**Affiliations:** 1 Department of Science (Microbiology), Assam down town University 497251, Guwahati, India; 2 Department of Microbiology, Shrimanta Shankaradeva University of Health Sciences 488260 , Guwahati, India; 3 Department of Science (Biotechnology), Assam down town University 497251, Guwahati, India; Nanchang University 47861, Nanchang, Jiangxi, China

**Keywords:** *Kosakonia *sp., Bacterial Genome, oral cavity

## Abstract

We report the draft genome sequence of *Kosakonia oralis* 282A-S69 isolated from the oral cavity of a habitual betel nut and tobacco user in Assam, India. The genome assembly is 4.8 Mb with a GC content of 56% and was assembled into five contigs with 100% completeness and 0.09% contamination. Average nucleotide identity and digital DNA-DNA hybridization analyses indicate that the isolate represents a potentially novel lineage within the genus *Kosakonia*.

## ANNOUNCEMENT

The genus *Kosakonia* (family Enterobacteriaceae) comprises Gram-negative facultatively anaerobic bacteria widely distributed in environmental and plant-associated niches, although several species have also been recovered from human clinical samples (1–3). Members of this genus were previously classified within *Enterobacter* but were reassigned based on multilocus sequence analysis and genome-based taxonomy (1). During a previous investigation of oral microbiota associated with intoxicant consumption in Northeast India (4), a bacterial isolate belonging to the genus *Kosakonia* was recovered from the oral cavity of a substance user.

Oral samples were collected, as previously described (4). Briefly, participants rinsed their mouths with sterile water for 20 s to remove loosely attached debris and transient microorganisms. The buccal mucosa, gingival surfaces, and tongue dorsum were then sampled using sterile cotton swabs under aseptic conditions. Swabs were transferred into sterile tubes and transported to the laboratory for microbiological analysis. The isolate obtained in this study was designated strain 282A-S69 and cultured aerobically on nutrient agar plates at 37°C.

Preliminary identification was performed using 16S rRNA gene sequencing, as previously described (4), which indicated that the isolate belonged to the genus *Kosakonia*.

Genomic DNA was extracted from freshly cultured bacterial cells using the HiPurA Bacterial Genomic DNA Purification Kit (MB505; HiMedia, India) according to the manufacturer’s instructions. DNA quality and concentration were evaluated using a Nanodrop spectrophotometer and agarose gel electrophoresis.

Whole genome sequencing was performed at Wipro Life Sciences Laboratory (Bengaluru, India) using the Illumina MiSeq platform. Sequencing libraries were prepared using the Nextera XT DNA Library Preparation Kit (Illumina, USA) and sequenced with paired-end reads (2 × 150 bp). A total of 61,571,624 paired-end reads were generated, providing approximately 99.9× genome coverage.

Raw sequencing reads were evaluated using FastQC v0.11.9, and adapter sequences and low-quality bases were removed using Trim Galore v0.6.7. *De novo* genome assembly was performed using SPAdes v3.15.5 (5). Assembly statistics were assessed using QUAST v5.2.0, and genome completeness and contamination were estimated using CheckM v1.2.4 (6). Unless otherwise specified, default parameters were used for all software tools.

The draft genome assembly consisted of 4,806,998 bp distributed across five contigs, with an N50 of 3,042,985 bp and an L50 of 1. The genome GC content was 56%, consistent with previously reported genomes of *Kosakonia* species (2, 7). Genome quality assessment indicated 100% completeness with 0.09% contamination. Genome annotation was performed using the NCBI Prokaryotic Genome Annotation Pipeline (PGAP) v6.4, which predicted 4,470 genes, including 4,346 protein-coding sequences and 87 RNA genes.

Functional annotation using BlastKOALA and antiSMASH v7.1.0 identified genes associated with metabolic functions and biosynthetic gene clusters including pyrroloquinoline quinone biosynthesis and enterobactin siderophore production (8, 9). Screening against the Comprehensive Antibiotic Resistance Database (CARD) v6.0.5 using the RGI tool detected primarily intrinsic resistance determinants typical of Enterobacteriaceae (10).

Taxonomic classification using Kraken2 v2.0.8-beta assigned the isolate to the genus *Kosakonia* (11). Phylogenomic analysis using the Type (Strain) Genome Server (TYGS) (accessed on 30 August 2025) identified *Kosakonia cowanii* JCM 10,956 as the closest validly described species (12). However, average nucleotide identity (ANI) analysis using EZBioCloud v20250421 yielded a value of 93.26%, below the commonly accepted species threshold of 95%–96% (13). Digital DNA–DNA hybridization (dDDH) analysis returned 50.9%, below the 70% species delineation threshold (14). These genomic metrics suggest that strain 282A-S69 represents a distinct genomic lineage within the genus *Kosakonia* and may represent a novel species.

## Data Availability

The draft genome sequence of *Candidatus Kosakonia oralis* sp. nov. has been deposited in GenBank under the accession number: JAROAU000000000.1, BioProject/BioSample: PRJNA935131/SAMN33298158), Genome assembly: GCF_029433855.1, SRA: SRP676478 and SSR run accession: SRR37203533.
